# Baltimore College of Dental Surgery

**Published:** 1873-10

**Authors:** 


### EDITORIAL, ETC.

---

*The Baltimore College of Dental Surgery.*—The thirty-fourth annual session of this, the oldest dental college in the world, will commence on the 15th of the present month, (October,) and continue until March, 1874.

The prospect for a class even larger than that of last session, is most encouraging; and an increased number of European students have already matriculated for the coming session.

During the past two months considerable improvements have been made in the college building, which, with those contemplated at an early day, will render it one of the most complete structures of the kind in the country. The infirmary and laboratory, each extending over the entire building, cannot be excelled for room, light and comfort. Every exertion will be made to maintain the high standard of this time honored institution.

